# Books and Magazines

**Published:** 1906-09

**Authors:** 


### Books and Magazines.

Morris' Anatomy. Prof. J. Playfair McMurrich, of the University of Michigan has assumed the American editorship and will himself contribute two articles.

For the first time in the history of the book American anatomists have been asked to contribute original articles and revise sections in a new edition of Morris' "Anatomy." By thus incorporating the results of recent investigations in American laboratories the book will have more of an international character, have a wider point of view, and be of greater use to teachers and students.

This edition of Morris will be, to large extent, a new book, modern in detail of both text and illustration, and in every respect representative of progressive methods and thought. It will be published by P. Blakiston's Son & Co.

---

THE AUTOTOXICOSES—THEIR THEORY, PATHOLOGY AND TREATMENT. By Heinrich Stern, Ph. M., M. D., New York, Professor of Special Medical Pathology and Therapy in the College of Physicians and Surgeons, Boston; Director of the Institute for Medical Diagnosis and Research in the City of New York; Physician-in-Chief, Philanthropic Hospital in the City of New York; Pediatrist and Pathologist, Misericordia Hospital and the Hartsdale Infirmary; Consulting Physician, Metropolitan and Red Cross Hospitals; Chairman Section on Pharmacology, American Medical Association; Permanent Member Medical Society, State of New York; Fellow of the New York Academy of Medicine, etc., etc. 12mo. 222 pages. Price, $1, postpaid. Chicago: G. P. Engelhardt & Co., 1906.

This book supplies a very evident want. Doctors know too little of the important subject with which it deals. Doubtless many of the cases of "heart-failure," "acute indigestion," etc., are caused by auto-intoxication, by poisons — unknown, perhaps, which are elaborated during digestion. It should be carefully read by all earnest practitioners of medicine.

---

PROPHYLAXIS AND TREATMENT OF INTERNAL DISEASES. By Frederick Forchheimer, M. D., Professor of Theory and Practice of Medicine and Clinical Medicine, Medical College of Ohio, University of Cincinnati, Ohio. Price, cloth, $5, net. D. Appleton & Co., Publishers, 436 Fifth Avenue, New York.

This is an eminently practical work, one which concerns itself diligently with the business in hand. It first lays down broad principles, then details the special applications of them, where possible; failing that, it indicates the proper direction for their application.

It is essentially a work of breadth. It is also essentially a work of experience. Free from dogmatism, there is the calm assurance of one to whom the path is familiar. In these days of therapeutic pessimism, it is refreshing to find a practical physician to whom the making of a correct diagnosis is but the beginning rather than the end of his craft.

Dr. Forchheimer undertook a difficult task, but we believe that he has given us a most excellent work,—one that will have a large sale to the general practitioner,—a book which has long been in demand. In short, this work is not the conventional manual of therapeutics consisting of a mere catalogue of things that may be done in a given case of diseases. It is rather a recovery of the missing chapters from the modern text-books of medicine. A necessary adjunct and companion to each and every other medical work.

---

BLAKISTON'S QUIZ-COMPENDS — A Compend of Materia Medica, Therapeutics and Prescription Writing, with Especial Reference to the Physiological Action of Drugs. Based on the Eighth Revision of the U. S. Pharmacopeia, including also Many Unofficial Remedies. By Samuel O. L. Potter, M. D., M. R. C. P., London. Formerly Professor of the Principles and Practice of Medicine in the Cooper Medical College of San Francisco; Author of "Materia Medica, Pharmacy, and Therapeutics," "Quiz-Compend of Anatomy," "Index of Comparative Therapeutics," and "Speech and Its Defects"; Late Major and Surgeon of Volunteers, U. S. Army. Seventh edition — revised and enlarged. Price, $1. P. Blakiston's Son & Co., 1012 Walnut St., Philadelphia, 1906.

We cordially recommend this book, especially to recent graduates and students.

A COMPEND OF OPERATIVE GYNECOLOGY, Based on Lectures in the Course of Operative Gynecology on the Cadaver at the New York Post-Graduate Medical School and Hospital. Delivered by William Seaman Bainbridge, M. D., Adjunct Professor of Operative Gynecology on the Cadaver, New York Post-Graduate Medical School and Hospital; Consulting Gynecologist, St. Mary's Hospital, Jamaica, L. I.; Consulting Gynecologist to St. Andrew's Convalescent Hospital, New York, etc. Compiled, with additional notes, in collaboration with Harold D. Meeker, M. D., Instructor in Operative Gynecology on the Cadaver, New York Post-Graduate Medical School and Hospital; Assistant, Department of Gynecology, Vanderbilt Clinic, College of Physicians and Surgeons, New York. Price not stated; about $1. The Grafton Press, Publishers, New York.

---

SURGICAL SUGGESTIONS — PRACTICAL BREVITIES IN SURGICAL DIAGNOSIS AND TREATMENT. By Walter M. Brickner, M. D., Chief of Surgical Department, Mount Sinai Hospital Dispensary, New York; Editor, *American Journal of Surgery,* and Eli Moschcowitz, M. D., Assistant Physician, Mount Sinai Hospital Dispensary, New York; Editorial Associate, *American Journal of Surgery.* Duodecimo; 60 pages. New York: Surgery Publishing Co., 1906. Cloth, 50 cents.

This little book is most novel, not only on account of the many original, terse and epigrammatic practical suggestions given, but its general appearance and attractive form. It contains 250 suggestions grouped under proper headings, and its contents is carefully indexed. While some of the items are familiar to the practical surgeon, they are presented in a manner that will impress them on the reader's memory. The book is bound in heavy cloth, stamped in gold, and the text is printed upon India tint paper with marginal headings in red. This book will be much appreciated by the general practitioner, not alone on account of the value of its contents, but as an artistic bit of book making.

---

ECZEMA: ITS CAUSE, DIAGNOSIS, AND TREATMENT; Embracing Many Points of Practical Importance, and Containing 146 Prescriptions, Illustrating Dosage in Local Applications. By Samuel Horton Brown, M. D., Assistant Dermatologist, Philadelphia Hospital, etc. Philadelphia: P. Blakiston's Son & Co., 1906. Price not stated (about $1).

If there is any one subject which the average doctor knows less about than any other, it is *eczema.* Buy this book and get next. The prescriptions alone are worth a dollar.

